# Rapid estimation of sugar release from winter wheat straw during bioethanol production using FTIR-photoacoustic spectroscopy

**DOI:** 10.1186/s13068-015-0267-2

**Published:** 2015-06-18

**Authors:** Georgios Bekiaris, Jane Lindedam, Clément Peltre, Stephen R. Decker, Geoffrey B. Turner, Jakob Magid, Sander Bruun

**Affiliations:** Department of Plant and Environmental Sciences, Faculty of Science, University of Copenhagen, Thorvaldsensvej 40, Frederiksberg, C DK-1871 Denmark; National Renewable Energy Laboratory, Biosciences Center, 15013 Denver West Parkway, Golden, Colorado 80401 USA

**Keywords:** Bioethanol production, FTIR-photoacoustic spectroscopy, Sugar release, Prediction, High-throughput assay, Pretreatment, Enzymatic hydrolysis, Advanced chemometrics

## Abstract

**Background:**

Complexity and high cost are the main limitations for high-throughput screening methods for the estimation of the sugar release from plant materials during bioethanol production. In addition, it is important that we improve our understanding of the mechanisms by which different chemical components are affecting the degradability of plant material. In this study, Fourier transform infrared photoacoustic spectroscopy (FTIR-PAS) was combined with advanced chemometrics to develop calibration models predicting the amount of sugars released after pretreatment and enzymatic hydrolysis of wheat straw during bioethanol production, and the spectra were analysed to identify components associated with recalcitrance.

**Results:**

A total of 1122 wheat straw samples from nine different locations in Denmark and one location in the United Kingdom, spanning a large variation in genetic material and environmental conditions during growth, were analysed. The FTIR-PAS spectra of non-pretreated wheat straw were correlated with the measured sugar release, determined by a high-throughput pretreatment and enzymatic hydrolysis (HTPH) assay. A partial least square regression (PLSR) calibration model predicting the glucose and xylose release was developed. The interpretation of the regression coefficients revealed a positive correlation between the released glucose and xylose with easily hydrolysable compounds, such as amorphous cellulose and hemicellulose. Additionally, a negative correlation with crystalline cellulose and lignin, which inhibits cellulose and hemicellulose hydrolysis, was observed.

**Conclusions:**

FTIR-PAS was used as a reliable method for the rapid estimation of sugar release during bioethanol production. The spectra revealed that lignin inhibited the hydrolysis of polysaccharides into monomers, while the crystallinity of cellulose retarded its hydrolysis into glucose. Amorphous cellulose and xylans were found to contribute significantly to the released amounts of glucose and xylose, respectively.

**Electronic supplementary material:**

The online version of this article (doi:10.1186/s13068-015-0267-2) contains supplementary material, which is available to authorized users.

## Background

Production systems for second generation biofuels produced from lignocellulosic biomass have been evolving in the last few decades in an attempt to reduce the environmental impact and sustainability issues arising from the wide-scale production and use of conventional biofuels [1]. Lignocellulosic biomass constitutes about 50 % of the world’s biomass [2], while it has been estimated that more than 442*10^9^ L of bioethanol can be produced per year from the lignocellulosic biomass left in the fields [3]. One of the challenges for the use of lignocellulosic biomass for bioethanol production is to develop cheap and efficient pretreatment methods that disrupt the lignocellulosic complex making the cellulose more amorphous as well as removing or degrading lignin [4]. The degradation of lignin makes plant biomass more susceptible to quick hydrolysis and increases the yields of monomeric sugars necessary for bioethanol production [5]. This increase in the yields of monomeric sugars results in the production of larger amounts of bioethanol.

However, even after pretreatment, differences in straw from different varieties or cultivars produced under different environmental conditions are still likely to prevail [6]. To select the best cultivars, it is desirable to assess the potential for sugar release after pretreatment and hydrolysis of a large number of cultivars. For this purpose, high-throughput screening methods have been developed [7–9]. The complexity of the required pretreatment and enzymatic hydrolysis of the biomass, as well as the cost per sample, are the main limitations of these techniques [10]. Near infrared spectroscopy (NIRS) has been adopted as a rapid analysis method that can predict the sugar release upon pretreatment and hydrolysis of groups of plant biomass [11–13]. Good prediction accuracy can be achieved using this technique, but it provides limited information about the chemical components that are associated with the propensity to release sugars. The reason for this is that the near infrared (NIR) spectra mostly reflect overtones and the combination bands of the chemical bonds, which are highly overlapping [14].

A large number of literature studies have provided insights on Fourier transform infrared (FTIR) spectra interpretation [15–17]. Attenuated total reflection FTIR (ATR-FTIR) spectroscopy has been adopted in the past to determine the changes that take place during the pretreatment of wheat straw [18], as well as the transformation of cellulose during the enzymatic hydrolysis for bioethanol production [19]. ATR-FTIR has also been used, in combination with advanced chemometrics, to predict the composition of pretreated softwood [20] as well as the glucan, xylan and other polysaccharide content of straw [21]. Only a limited number of attempts have been made to apply mid-infrared spectroscopy in the prediction of fermentable sugars from pretreated biomass [16, 22, 23]; there have been no previous attempts to correlate the FTIR or Fourier transform infrared photoacoustic (FTIR-PA) spectra of non-pretreated biomass with their potential sugar release. FTIR-PAS arises from combining traditional FTIR and a photoacoustic detector (PA). The measurement of the absorbed radiation is directly proportional to the heat wave produced after the interaction of the sample with the IR radiation. In this way, the measurement remains unaffected by the redistribution of the light due to scattering effects or diffraction processes [24–26].

Therefore, the aim of the present study was to use FTIR-PAS for the characterisation of winter wheat straw and identification of chemical structures related to sugar release and to develop calibrations predicting potential sugar release from FTIR-PA spectra.

## Results and discussion

### Spectroscopic analysis

**Fig. 1:**
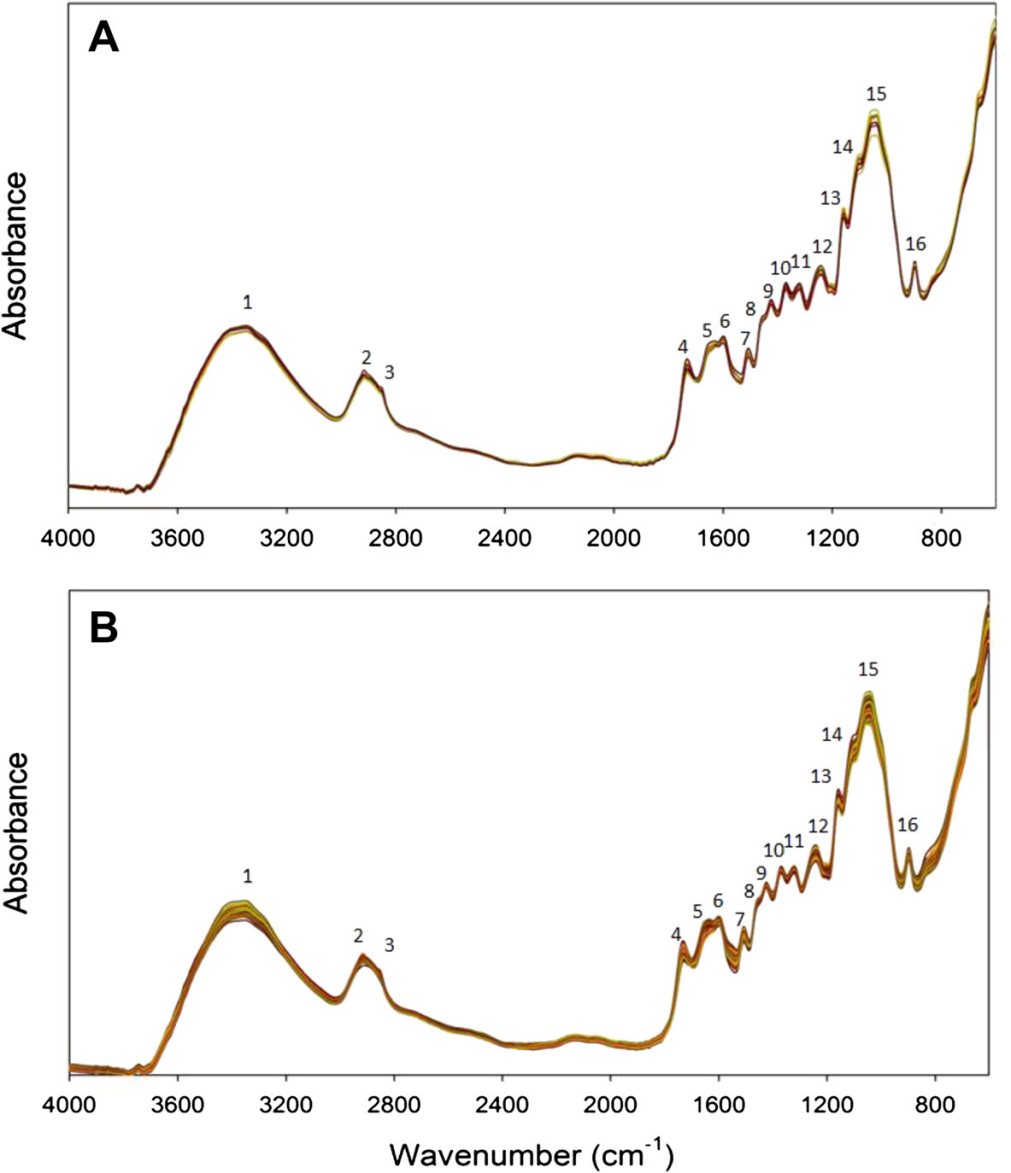
FTIR-PA spectra of winter wheat straw. **a** Spectra averaged across different locations (nine spectra). **b** Spectra averaged across different wheat straw varieties (203 spectra)

The averaged spectra of each site and variety were characterised by common peaks with slightly different absorption intensities (Fig. 1a, b). The different peaks correspond to fundamental molecular stretching and bending vibrations of different chemical groups in the samples (Table 1). The broad peak centred at 3380 cm^−1^ (peak 1) can be assigned to water or lignin from wood samples, while the peak at 2920 cm^−1^ (peak 2) and the shoulder at 2850 cm^−1^ (peak 3) correspond to aliphatics. Ciolacu et al. [27] observed a shift in this peak from 2900 cm^−1^ for pure cellulose to 2920 cm^−1^ for the amorphous cellulose. In the fingerprint region (1800–600 cm^−1^) of the spectrum, strong absorption was observed at 1735 cm^−1^ (peak 4), which, as the shoulder at 1460 cm^−1^ (peak 8), correspond to xylans. The peak at 1650 cm^−1^ (peak 5), which revealed a diversification in the absorption intensity, corresponds either to carboxylates or the absorbed water; therefore, the difference in the absorption intensity probably indicated different contents of carboxylates, as all samples were dried following the same procedure. The peaks at 1600 (peak 6) and 1510 cm^−1^ (peak 7) are associated with lignin. The IR absorption at 1429 cm^−1^ (peak 9) corresponds to lignin or crystalline cellulose, while the peak at 1370 cm^−1^ (peak 10) can be assigned to cellulose and hemicellulose. Ciolacu et al. [27] observed a positive correlation of crystalline cellulose with both regions (1429 and 1370 cm^−1^) for various materials, while both of them seem to be absent in amorphous cellulose or replaced by a strong peak shifted at 1400 cm^−1^. The relatively strong peak that was visible at 1320 cm^−1^ (peak 11) could be part of either the peak at 1335 cm^−1^ observed by Pandey and Pitman [28] corresponding to the C-H vibration of cellulose, hemicellulose, lignin, or the peak at 1310 cm^−1^ observed by Sills and Gossett [16] corresponding to the CH_2_ wagging in cellulose and hemicellulose. The relatively broad peak at 1240 cm^−1^ (peak 12) could be assigned to xylans, while the peak at 1160 cm^−1^ (peak 13) corresponds to cellulose and hemicellulose. According to Ciolacu et al. [27], while this peak is observed in the FTIR spectra of original cellulose, it is absent in the spectra of the amorphous form of cellulose. Both peaks at 1111 cm^−1^ (peak 14) and 1053 cm^−1^ (peak 15) correspond to crystalline cellulose, while the peak at 898 cm^−1^ (peak 16) can be assigned to amorphous cellulose.

**Table 1:** Most important absorption bands of the mid-infrared spectra of winter wheat straw

Peak no. in Fig. 1	Wavenumber (cm^−1^)	Vibration	Assignment
1	3380	O-H stretching of bonded and non-bonded hydroxyl groups	Included water ^i^; lignin ^e^
2	2920	Asymmetric C-H stretching	Aliphatic methylene ^b, e, g, k^
3	2850	Symmetric C-H stretching	
4	1735	Un-conjugated C = O stretching	Xylan (hemicellulose) ^a–c, e, f, i, j^
5	1650	O-H bending	Absorbed water ^a, i^
		Conjugated C-O stretching	Carboxylates ^a, i^
6	1600	Aromatic ring vibration	Lignin ^a, c, g, j^
		C = C skeletal vibration	
		C = O stretching	
7	1510	Aromatic ring vibration	Lignin ^a, c, d, g, j^
8	1460	C-H deformation	Lignin ^a, c^; xylan ^j^
9	1429	C-H deformation	Lignin ^a, g, I, j^
		CH_2_ scissoring	Crystalline cellulose ^b^
10	1370	Symmetric C-H deformation	Crystalline cellulose ^k^; hemicellulose ^a, i^
11	1320	C-H vibration	Cellulose, hemicellulose, lignin ^a^
		CH_2_ wagging	Cellulose, hemicellulose ^c^
12	1240	C-O stretching	Xylan (hemicellulose) ^a, i^
13	1160	C-O-C asymmetric stretching	Crystalline cellulose ^b, j, k^; hemicellulose ^a, h^
14	1111	In-plane ring stretching	Crystalline cellulose ^b, h, j^
15	1053	C-O stretching	Crystalline cellulose; hemicellulose ^a, b, h, k^
16	898	C-O-C stretching	Amorphous cellulose ^k^

^a^Pandey and Pitman [28]

^b^Gwon et al. [36]

^c^Sills, Gossett [16]

^d^Gollapalli et al. [22]

^e^Xu et al. [15]

^f^Kristensen et al. [18]

^g^Merk et al. [42]

^h^Corgie et al. [19]

^i^Cui et al. [43]

^j^Chen et al. [44]

^k^Ciolacu et al. [27]

### Sugar release

The high-throughput pretreatment and enzymatic hydrolysis (HTPH) measurements of the samples shown in Table 2 revealed a range in the sugar yield from 0.28 to 0.59 g g^−1^ of dry matter (dm) for total sugars, 0.14 to 0.50 g g^−1^ dm for glucose and 0.06 to 0.29 g g^−1^ dm for xylose release (mean values of 0.42, 0.23 and 0.19 g g^−1^ dm for total sugar, glucose and xylose release, respectively). The high-yielding straw samples released approximately double the amount of total sugar in comparison to the low-yielding samples, indicating a substantial span in bioethanol potential. The low standard deviation of the laboratory method (SDL) of 0.024 g g^−1^ dm for total sugar, 0.016 g g^−1^ dm for glucose and 0.010 g g^−1^ dm for xylose indicated that the reproducibility of the HTPH assay was high. Explaining the causes for variability of the ethanol potential, as undertaken by Lindedam et al. [6], was beyond of the scope of this study, but generally speaking, annual variation and the effect of cultivar, site and environment are highly influential.

**Table 2:** Experiments from which straw samples has been collected

Experiment	Year	Locations	Treatments	Field replicates	Number of samples
Variety testing	2006	Abed	106 modern Northern European varieties	1	206
		Sejet			
Variety testing	2006	Abed	20 modern Northern European varieties	1	79
		Holstebro			
		Sejet			
		Tystofte			
Variety testing	2007	Abed	20 modern Northern European varieties	4	317
		Holstebro			
		Sejet			
		Tystofte			
Old varieties	2007	Taastrup	102 old varieties released to the market in the period from 1902 to 1990	2	167
Old varieties	2008	Taastrup	102 old varieties released to the market in the period from 1902 to 1990	2	201
Fertilisation experiment	2008	Rothamsted	1 variety with 19 different fertiliser applications of organic and inorganic fertilisers	3	57
Variety testing	2008	Holstebro	10 modern Northern European varieties	4	80
		Søtoften			
Full scale	2008	Fyn	5 varieties	1	10
		Holstebro			
Maturity degree	2008	Hornsherred	2 varieties at 3 harvests (3 weeks before maturity, maturity stage, 3 weeks after maturity)	1	5
Total					1122

### Prediction of sugar release

**Fig. 2:**
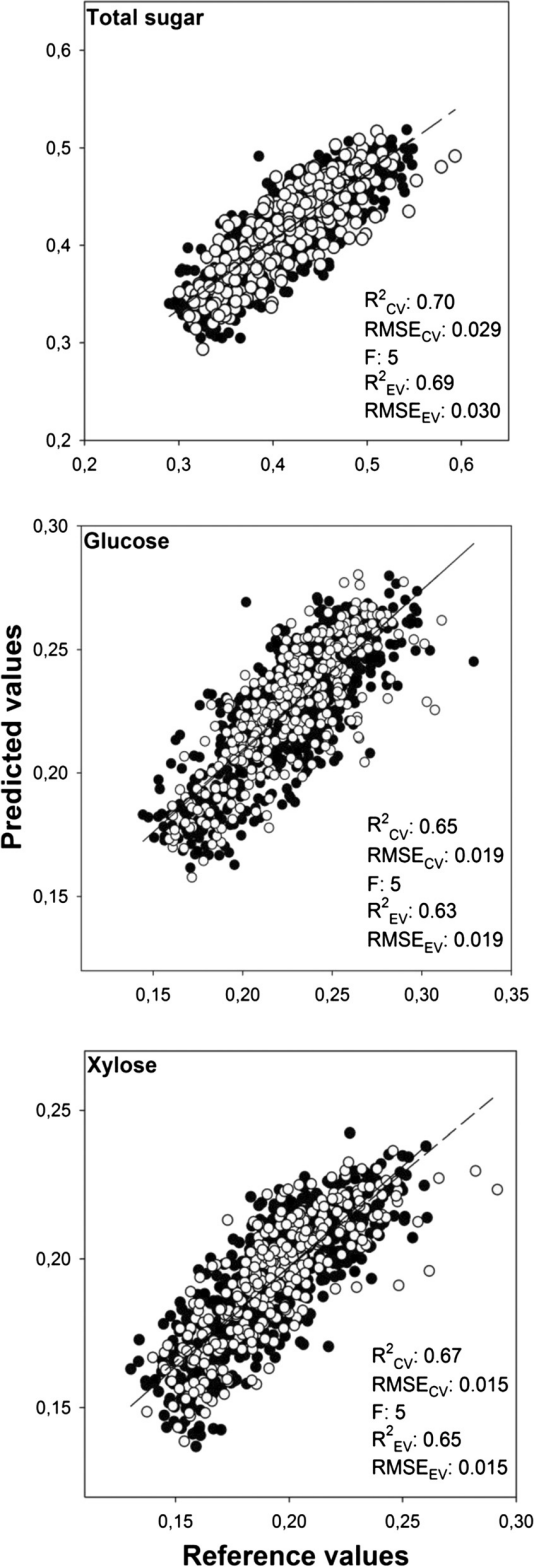
Measured vs. predicted values of sugar release. Correlation between reference (measured) and predicted sugar release (in g g^−1^ dm) in terms of total sugar (glucose plus xylose), glucose and xylose (cross-validation results; *black dots*, *solid regression line*, external validation results: *white dots*, *dashed regression line*). (*R* ^2^ coefficient of determination, *RMSE* root-mean-square value, *CV* cross-validation data set, *EV* external validation data set, *F* number of factors used in calibration)

The different transformation methods of the spectra did not considerably improve the accuracy of the predictions of sugar release (Table 3) and only the first derivative transformation resulted in slightly better predictions than the smoothed and normalised spectra. Both first and second derivative transformations needed a lower number of components (factors) for the predictions, which indicated that the transformation reduced some information that was of little predictive value (Table 3). In all cases, a fair prediction of the potential total sugar, glucose and xylose release was obtained, and the *R*^2^ (coefficient of determination) values of the predictions for the external validation (EV) data set using the smoothing/normalisation transformation were 0.69 for total sugar, 0.63 for glucose and 0.65 for xylose. The root-mean-square error (RMSE) for the same predictions were 0.030, 0.019 and 0.015 g g^−1^ dm, respectively (Table 3, Fig. 2), while the ratio of RMSE_EV_ to SDL was 1.25, 1.18 and 1.45. In addition to the low RMSE, the differences between cross-validation and the external validation results were quite small, which indicated that the calibrations were robust. These results proved the potential use of calibrations based on FTIR-PAS for the prediction of sugar release from wheat straw. Considering the wide variation in genetic material and environmental conditions during growth, it is reasonable to assume that the model may be applied to other winter wheat straw materials. Applicability of these calibrations in other types of plant biomass have not been tested, but it could be feasible since the right regions of the spectrum, corresponding to compounds relevant to the sugars, were taken into account in the calibrations (see section Analysis of regression coefficients).

**Table 3:** Different spectral transformations. Effect of the different preprocessing of the spectra on the prediction of total sugar, xylose and glucose release during bioethanol production (R^2^ coefficient of determination, RMSE root-mean-square error, CV cross-validation data set, EV external validation data set, F number of factors used in calibration)

Preprocessing	Total sugar					Glucose					Xylose				
	*R* ^2^		F	RMSE (g g^−1^ dm)		*R* ^2^		F	RMSE (g g^−1^ dm)		*R* ^2^		F	RMSE (g g^−1^ dm)	
	CV	EV		CV	EV	CV	EV		CV	EV	CV	EV		CV	EV
Savitzky-Golay smoothing (seven points)	0.70	0.69	5	0.029	0.030	0.65	0.63	5	0.019	0.019	0.67	0.65	5	0.015	0.015
Normalisation by mean															
Savitzky-Golay first derivative	0.71	0.70	4	0.029	0.030	0.65	0.64	4	0.019	0.019	0.68	0.66	4	0.014	0.015
(second order polynomial, seven smoothing points)															
Savitzky-Golay second derivative	0.71	0.63	4	0.029	0.030	0.66	0.55	4	0.018	0.021	0.70	0.60	5	0.014	0.016
(second order polynomial, seven smoothing points)															
Savitzky-Golay smoothing (seven points)	0.71	0.69	5	0.029	0.030	0.66	0.63	5	0.018	0.019	0.67	0.65	5	0.015	0.015
Standard normal variate (SNV)

A number of other studies have used mid-infrared spectroscopy to predict potential ethanol production from biomass. Gollapalli et al. [22] obtained correlations between glucose yield and the diffuse reflectance infrared Fourier transform (DRIFT) spectra, with *R*^2^ values ranging between 0.65 and 0.71 for the different hydrolysis time points of initial rice straw, while the *R*^2^ values of xylose concentration ranged between 0.47 and 0.50. Sills and Gossett [16] were able to explain a larger fraction of the variation during the prediction of glucose and xylose release in a sample set of 24 pretreated and hydrolysed biomass samples (six different plant materials, four different pretreatments with NaOH) using the fingerprint region (1800–800 cm^−1^) of the ATR-FTIR spectra obtained. The obtained *R*^2^ values of 0.86 and 0.84 for the glucose and xylose content, respectively, were higher than this study’s values of 0.63 and 0.65. However, the RMSE values they obtained were 0.078 g g^−1^ dm for glucose and 0.093 g g^−1^ dm for xylose release, which are higher than the 0.019 and 0.015 g g^−1^ dm, respectively, that were obtained in the present study. The high uniformity in this study’s sample set (all the straw samples being wheat straw from a relatively small geographical region) meant that the variation in the sample set was small and supported the lower RMSE values. In addition, the use of an external validation data set in the present study can provide more certainty about the predictive power of the model and eliminate the possibility of an overestimation of *R*^2^ values. Martin et al. [23] developed a model predicting the cell wall digestibility of *Sorghum bicolor* biomass using the fingerprint region (1800–850 cm^−1^) of the obtained ATR-FTIR spectra, with a high *R*^2^ value of 0.94 and an RMSE of 0.64 μg mg^−1^ dry weight h^−1^. In their study, the samples were collected at different developmental stages, resulting in high variable digestibility between the samples. This could explain the high predictive power of their model. The model developed in the present study predicting the total sugar release resulted in a lower *R*^2^ value, but the samples were also displaying less variability with all samples stemming from mature wheat straw. Castillo et al. [29] applied PLSR to develop a model predicting the ethanol production from *Eucalyptus globulus* pulp using mid-infrared spectroscopy. They obtained an *R*^2^ value of 0.92 with an RMSE of 1.9 g L^−1^ for the calibration sample set, while the validation of the model by an external validation set gave an *R*^2^ value of 0.60. The big difference in the *R*^2^ values between calibration and external validation sample sets may indicate the overestimation in the calibration.

NIR spectroscopy has also been used on a number of occasions to predict sugar release or digestibility of biomass samples. Lindedam et al. [12] predicted the sugar release of untreated air-dried wheat straw and achieved *R*^2^ values of 0.56 for the total sugar release, 0.44 for the glucose and 0.69 for the xylose release with RMSE values of 0.014, 0.010 and 0.005 g g^−1^ dm, respectively. Bruun et al. [30] performed partial least squares (PLS) calibration in order to predict the degradability of wheat straw obtaining an *R*^2^ value of 0.72 and an RMSE of 1.4 % using untreated wheat straw from two different sites. These values are difficult to compare with ours because of different reference methods and sample variability, but they seem to be in the same range and thus indicate that the predictive power of NIR is similar to FTIR-PAS.

A few studies have also been using spectroscopic methods to predict the results of biomass compositional analysis. Tucker et al. [20] applied PLS analysis to develop a model predicting the glucan and xylan content from 35 ATR-FTIR spectra of forest thinning and softwood sawdust (hemlock, Sitka spruce and red cedar). Tamaki and Mazza [21] developed models predicting the glucan and xylan content of wheat and triticale using ATR-FTIR spectra. These studies generally obtained very high predictive power and precision. This may reflect the fact that predictions of the total amount of the specific sugars are easier than predicting the digestible parts. This may be explained by the fact that total cellulose and xylan appears in the spectra as specific bands whereas the digestible amount of the same components depends on a range of other chemical components that may impede the enzymatic hydrolysis of cellulose and xylan.

### Analysis of regression coefficients

#### Regression coefficients of total sugar prediction

**Fig. 3:**
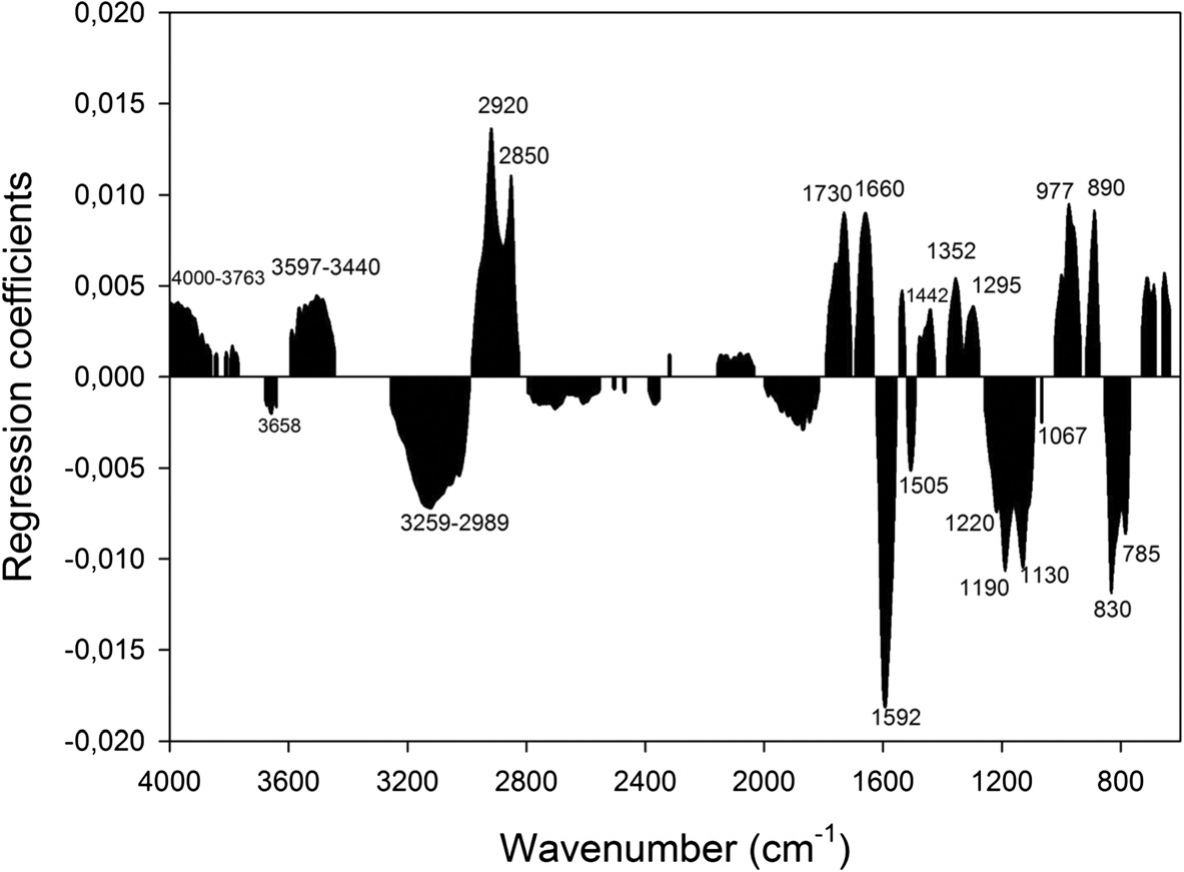
Regression coefficients from the prediction of total sugar release. Spectral regions with a significant contribution in the prediction of total sugar release after the pretreatment and enzymatic hydrolysis of wheat straw and during bioethanol production

Positive regression coefficients (Fig. 3) were obtained in the region of 3597–3440 cm^−1^ of the spectrum dominated by the stretching vibration of the O-H bond in various compounds, making an interpretation of this region difficult. Nevertheless, Ciolacu et al. [27] suggest that this broad peak is observed in both crystalline and amorphous forms of cellulose, but with a shift towards higher wavenumbers (around 3440 instead of 3350 cm^−1^) for amorphous cellulose. The strong positive association with fermentable sugars, which was observed at 2920 and 2850 cm^−1^, corresponds to the aliphatic methylene and is present in the spectrum of amorphous cellulose. The regions at 1730 and 1660 cm^−1^ are attributed to hemicelluloses and carboxylates. Additionally, a positive association with the sugar release was observed in the regions at 1442 and 1352 cm^−1^. According to Liang, Marchessault [31, 32], these regions correspond to the O-H bending in-plane vibration (1442 cm^−1^) and the C-H bending vibration (1352 cm^−1^) of cellulose and hemicellulose. The positively associated region, centred around 1295 cm^−1^, can be attributed to CH_2_ wagging [16] in cellulose and hemicellulose or the C-H deformation in hemicelluloses [33]. Finally, both regions at 977 and 890 cm^−1^ are associated with C-O-C stretching at the β-(1 → 4)-glycosidic linkages of amorphous cellulose [27]. The interpretation of the positive regression coefficients in this study revealed a strong correlation of sugar release with amorphous cellulose and hemicellulose.

The broad negative associated regions between 3259 and 2989 cm^−1^ correspond to the O-H stretching vibration of various compounds and, as mentioned earlier, their interpretation is difficult. Fengel [34] asserts that the region of the IR spectrum between 3200 and 3700 cm^−1^ arises from the intra- and inter-molecular O-H vibrations of crystalline cellulose. The crystalline forms of cellulose appear to be more resistant to enzymatic hydrolysis [35]; therefore, it was expected to be negatively associated with the sugar release. The strongly negatively associated regions at 1592 and 1505 cm^−1^ are attributed to lignin, which has been found to play an inhibitory role in the hydrolysis of cellulose and hemicellulose into fermentable sugars [36]. Additionally, the region at 1220 cm^−1^ can be assigned either to the C-C/C-O stretching vibration in lignin [37] or the C-O-H in-plane bending vibration in crystalline cellulose [38]. Finally, the regions at 1190, 1130 and 1067 cm^−1^ are associated with crystalline cellulose, while there is not as much information related to the regions under 830 cm^−1^. Liang and Marchessault [31] suggested that the regions near 740 and 800 cm^−1^ are assigned to the CH_2_-rocking vibration of crystalline cellulose. The interpretation of the negative regression coefficients in this study revealed a negative correlation of sugar release with regions related to lignin and crystalline cellulose. This is not surprising as lignin plays an inhibitory role in the hydrolysis of celluloses and hemicelluloses. Furthermore, the hydrolysis of crystalline cellulose is much slower than amorphous cellulose, as the adsorption of the enzymes necessary for hydrolysis declines with increasing cellulose crystallinity [39].

#### Regression coefficients of x*ylose and glucose prediction*

**Fig. 4:**
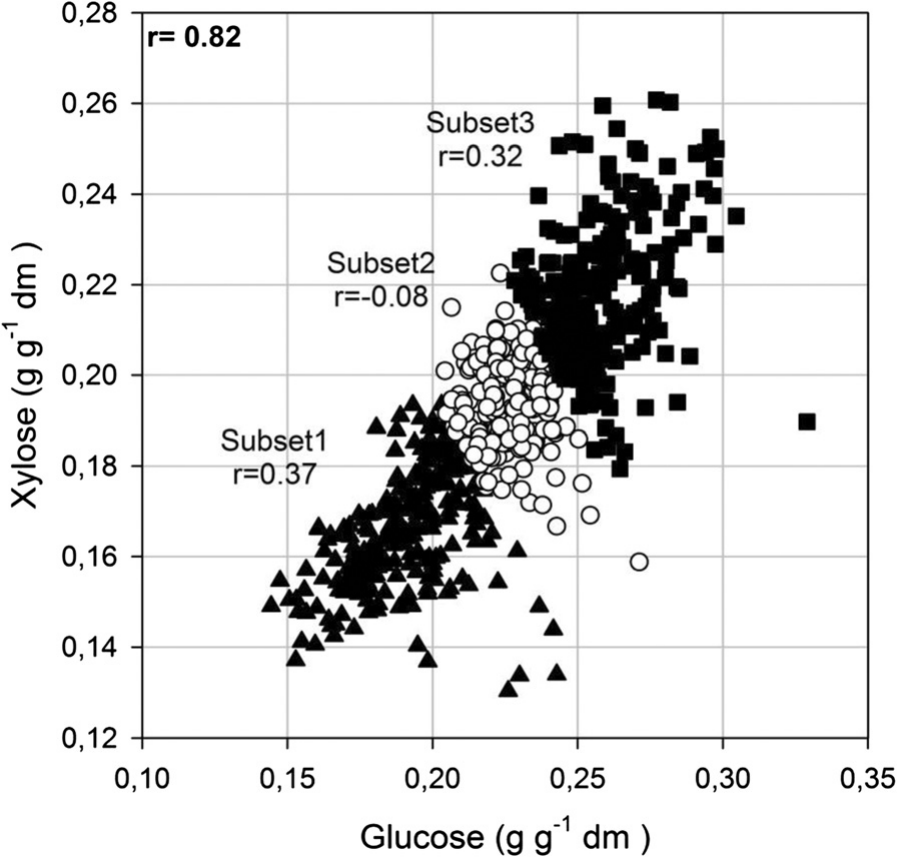
Xylose vs. glucose release after the pretreatment and enzymatic hydrolysis. Correlation coefficients (*r*) of the measured glucose and xylose yields (in g g^−1^ dm) in the full calibration set (713 samples) and the three smaller subsets (of 237 samples each). *Triangles* subset 1, *circles* subset 2, *squares* subset 3

**Fig. 5:**
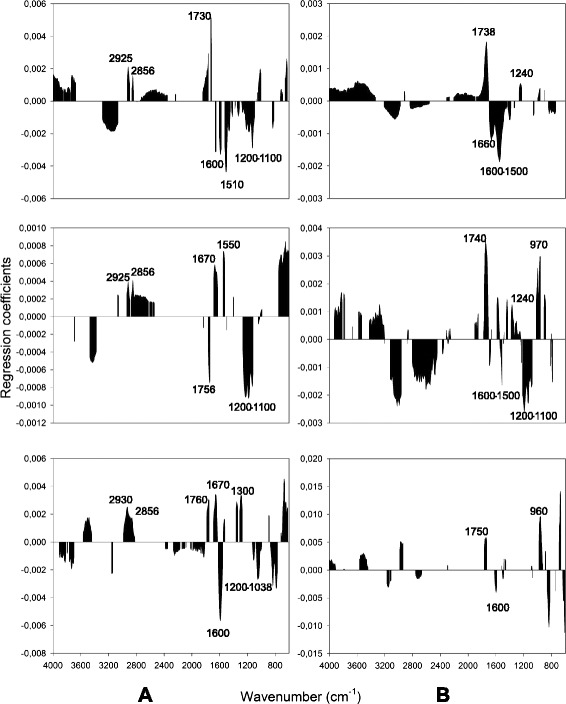
Regression coefficients from the prediction of glucose and xylose release. Spectral regions with a significant contribution in the prediction of glucose (**a**) and xylose (**b**) release during bioethanol production based on each of three subsets; *top* (subset 1), *middle* (subset 2), *bottom* (subset 3)

The high correlation (*r* = 0.82) of the measured glucose and xylose yields could mean that the developed calibration model for each sugar monomer might be built on regions of the spectrum determining the other variable. This fact could explain why the same regions of the spectrum were used for the prediction of total sugar, glucose and xylose release (Additional file 1: Figure S1). The division of the calibration set into three smaller subsets led to a decrease in the correlation between the measured glucose and xylose yields from 0.82 in the full calibration set to 0.37, 0.08 and 0.32 in each of the three subsets, respectively (Fig. 4). The partial least square regression (PLSR) analysis, which was performed on each subset, revealed the spectral regions that were associated with the release of each sugar monomer (Fig. 5).

The differences in the regression coefficients obtained for the prediction of glucose release between the three sample subsets (Fig. 5a) were more obvious than those of xylose (Fig. 5b). Positive regression coefficients at the regions around 2920 and 2850 cm^−1^ (aliphatics/amorphous cellulose) appeared in all subsets (Fig. 5a), while a positive association with the region at 1670 cm^−1^ (carboxylates) was present in two of the subsets. The region between 1200 and 1100 cm^−1^, which is associated with crystalline cellulose, displayed negative regression coefficients in all subsets, indicating that this region contributed to glucose prediction to a limited extent. Additionally, the region between 1600 and 1500 cm^−1^ (associated with lignin) displayed negative regression coefficients in two of the subsets. Both regions are therefore related to the restriction of cellulose hydrolysis and consequently, the release of glucose.

In contrast, the regression coefficients obtained for xylose prediction were fairly similar, regardless of which of the three sample subsets was used (Fig. 5b). Xylose release was found to be positively associated with the region around 1740 cm^−1^ in all subsets and the region around 1250 cm^−1^ in two of the subsets. Both of them are assigned to the xylans of hemicelluloses, which are built up by xylose monomers and are easily hydrolysable [36]. Negative regression coefficients were obtained in the region between 1500 and 1600 cm^−1^, which are assigned to lignin. This was expected in all subsets as lignin inhibits the hydrolysis of hemicelluloses.

The regions at 1730 (hemicelluloses) and 970 cm^−1^ (amorphous cellulose), which were present in the regression coefficients for glucose and xylose prediction, respectively, revealed that some correlation between the two sugar monomers remained, even after subdivision of the calibration set.

## Conclusions

This study established that FTIR-PAS can be used to predict the bioethanol potential from wheat straw and in addition provide structural information on the chemical compounds involved in saccharification. The predictions of total sugar, glucose and xylose release after pretreatment and enzymatic hydrolysis of wheat straw can be characterised as fair (coefficient of determination ranging between 0.64 and 0.70) and accurate (RMSE value ranging between 0.015 and 0.030 g g^−1^ dm and RMSE to SDL ratio between 1.18 and 1.45), especially considering the low variability of the sample set in this study caused by the fact that all samples stemmed from mature wheat straw.

The interpretation of the regression coefficients used for the predictions allowed the detection of compounds that contribute to the release of sugars and compounds that do not contribute or even inhibit hydrolysis. As expected, lignin was found to inhibit the hydrolysis of polysaccharides into monomers, while the crystallinity of cellulose might delay its hydrolysis into glucose. On the other hand, amorphous cellulose and xylans were found to contribute significantly to the released amounts of glucose and xylose, respectively.

## Materials and methods

### Sample collection and preparation

A total of 1122 wheat straw samples were collected from nine different locations in Denmark and one location in the United Kingdom (Table 4) from 2006 to 2010. The samples were collected from ongoing experiments with different wheat varieties, fertiliser treatments and harvesting times. The experiments included a total of 203 different wheat varieties. An overview of the origin of samples in terms of experiments, sites and treatments is given in Table 2.

**Table 4:** Experiment locations where wheat straw samples has been collected

Site name	Country	Coordinates
Abed	Denmark	54° 49' 40" N, 9° 55' 22" E
Sejet	Denmark	55° 49' 12" N, 11°19' 31" E
Holstebro	Denmark	56° 24' 5" N, 8° 38' 22" E
Tystofte	Denmark	55° 15' 9" N, 11° 20' 14 E
Taastrup	Denmark	55° 40' 36" N, 12° 18' 10" E
Rothamsted	United Kingdom	51° 48' 24" N, 0° 21' 49" W
Søtoften	Denmark	56° 14' 49" N, 10° 6' 1" E
Fyn	Denmark	55° 18' 29" N, 10° 22' 36" E
Hornsherred	Denmark	55° 78' 49" N, 11° 96' 59" E

From all but one experiment in Denmark, mature air-dried straw (approximately 7 % moisture) was sampled from the experimental pots after the grain had been harvested by a combine harvester cutting the straw and leaving it in the field. Approximately 80 g of straw was collected representatively from each plot, as described by Lindedam et al. [12] and stored at ambient temperature. Material from the experiment with different harvest times was collected by hand three weeks before maturity, at maturity and three weeks after. The plants were cut 5–7 cm from the soil, and the grain was removed from the samples before being stored at ambient temperature. Material from the UK was collected as described by Murozuka et al. [40]. Subsequently, all straw samples were ground on a cyclone mill (President, Holbaek, Denmark) mounted with a 1-mm screen.

### Determination of sugar release

Determination of potential sugar release was carried out at the National Renewable Energy Laboratory (NREL) in Denver, Colorado using a slightly modified method [41] compared to the one described by Selig et al. [9]. Briefly, 2 % dm solids (5.0 ± 0.3 mg in 250 μL of de-ionised H_2_O) were pretreated in triplicate in a 96-well plate in a steam chamber for 17.5 min at 180 °C, with heat-up and cool-down phases of approximately 52 sec and 1.5 min (to reach 120 °C), respectively [9]. Hydrolysis was started by loading total enzyme protein on dry biomass at 70 mg g^−1^ dm of Cellic® CTec2 (Novozymes, Bagsværd, Denmark). After enzymatic hydrolysis at 50 °C for 70 h, release of glucose and xylose was measured by a glucose oxidase/peroxidase assay and a xylose dehydrogenase assay, respectively (Megazyme International Ireland, Wicklow, Ireland). Total sugars were the calculated values of glucose plus xylose in each sample. Any sugars added with the enzyme mix were accounted for with enzyme-only blanks in every plate.

### Fourier transform infrared photoacoustic spectroscopy (FTIR-PAS)

No pretreatment of the ground samples was performed prior to the spectroscopic analysis, apart from oven drying at 70 °C for 48 hours. The FTIR-PAS spectra were recorded using a Nicolet 6700 (ThermoScientific, USA) spectrometer equipped with a PA-301 photoacoustic detector (Gasera Ltd, Finland). During the measurement, there was a purging flow with helium gas to reduce the noise caused by moisture evaporating from the samples. The samples were packed in small ring cups of 10-mm diameter and inserted into the PA detector. For each sample, 32 scans in the mid-infrared region between 4000 and 600 cm^−1^ at a resolution of 4 cm^−1^ were recorded and averaged. Subsequently, the spectra were smoothed by the Savitzky-Golay algorithm [42] using three points on each side (total window of seven smoothing points) and a zero polynomial, and normalised by the mean using The Unscrambler v.10.3 software (CAMO software, Oslo, Norway).

### Multivariate analysis

PLSR was used to calibrate models predicting glucose and xylose release from the FTIR-PA spectra. Different preprocessing of the spectra were performed in an attempt to obtain better predictions (Table 3). Prior to the PLSR analysis, 54 outliers were removed to increase the model’s stability. The selection of the outliers was based on the observation of the Residual vs. Hotelling-T^2^ distribution implemented in the software. In order to avoid a possible overestimation, the sample set was divided into a calibration set that contained two thirds of the samples (713 samples) and a smaller external validation set with randomly selected samples from all varieties and sites (355 samples). The calibration set was used to develop calibration models in which the optimal number of components was chosen based on a leave-one segment-out cross-validation using 10 segments of 71 samples. More stable and robust models were achieved by the variable selection method, known as Martens’ uncertainty test [43]. Subsequently, the samples of the external validation set were used to evaluate the robustness of the developed model. The Unscrambler v.10.3 software (CAMO, Oslo, Norway) was used for all calibrations.

After the models had been developed, the regression coefficients were interpreted in order to understand which chemical components were correlated with xylose and glucose release respectively. However, glucose and xylose turned out to be highly correlated (*r* = 0.82). This essentially meant that the regions of the spectrum were not uniquely related to the monomeric sugar that the model was predicting. For example, a model predicting glucose may have high regression coefficients in a region that is related to xylose because xylose is correlated with glucose. In order to be able to identify regions that are uniquely responsible for predicting glucose and not derived from the correlation with xylose, three datasets were produced to reduce the correlation between glucose and xylose. Calibration models were subsequently made predicting glucose and xylose for the data in each of these datasets, and the regression coefficients for these datasets were inspected and interpreted.

The performance of the PLSR-calibrations was determined by the coefficient of determination (*R*^2^):

$$ {R}^2 = \frac{{\displaystyle {\sum}_i}{\left({y}_i-{f}_i\right)}^2}{{\displaystyle {\sum}_i}{\left({y}_i-\overline{y}\right)}^2} $$

where *y*_i_ represents the observed values and *f*_i_ the predicted values.

The closer the *R*^2^ is to 1, the better the fit of the reference values (*y*_i_) to the regression line.

The accuracy of the calibrations was determined by the root-mean-square error (RMSE) (in g g^−1^ dm):

$$ RMSE=\sqrt{{\displaystyle \sum_{i=0}^n{\left({f}_i-{y}_i\right)}^2/n}} $$

In addition, the standard deviation of the laboratory method (SDL) was calculated:

$$ SDL=\sqrt{\frac{{\displaystyle {\sum}_{i=1}^n{\displaystyle {\sum}_{j=1}^m{\left({y}_{ij}-{\overline{y}}_j\right)}^2}}}{m*n-1}} $$

where *i* is the laboratory replicate out of *m* replicates and *j* is the individual sample out of *n* samples.

The closer the ratio of RMSEEV over SDL is to 1, the better the predictive power of the model to the reference measurements.

## Additional file

Additional file 1:
**Regression coefficients from the prediction of glucose and xylose release before the division of the calibration set into three smaller subsets.** Spectral regions with a significant contribution in the prediction of glucose (A) and xylose (B) release during bioethanol production.

## Abbreviations

ATR-FTIR: attenuated total reflection FTIR
DRIFT: diffuse reflectance infrared Fourier transform
EV: external validation
FTIR: Fourier transform infrared
FTIR-PAS: FTIR-photoacoustic spectroscopy
HTPH: high-throughput pretreatment and enzymatic hydrolysis
NIR: near infrared
PLSR: partial least square regression
RMSE: root-mean-square error
SDL: standard deviation of the laboratory method
