# Supplementary figures and images for: Rapid estimation of sugar release from winter wheat straw during bioethanol production using FTIR-photoacoustic spectroscopy
Source: Biotechnol Biofuels. 2015 Jun 18;8:85. doi: 10.1186/s13068-015-0267-2 (PMC4479319; doi:10.1186/s13068-015-0267-2)

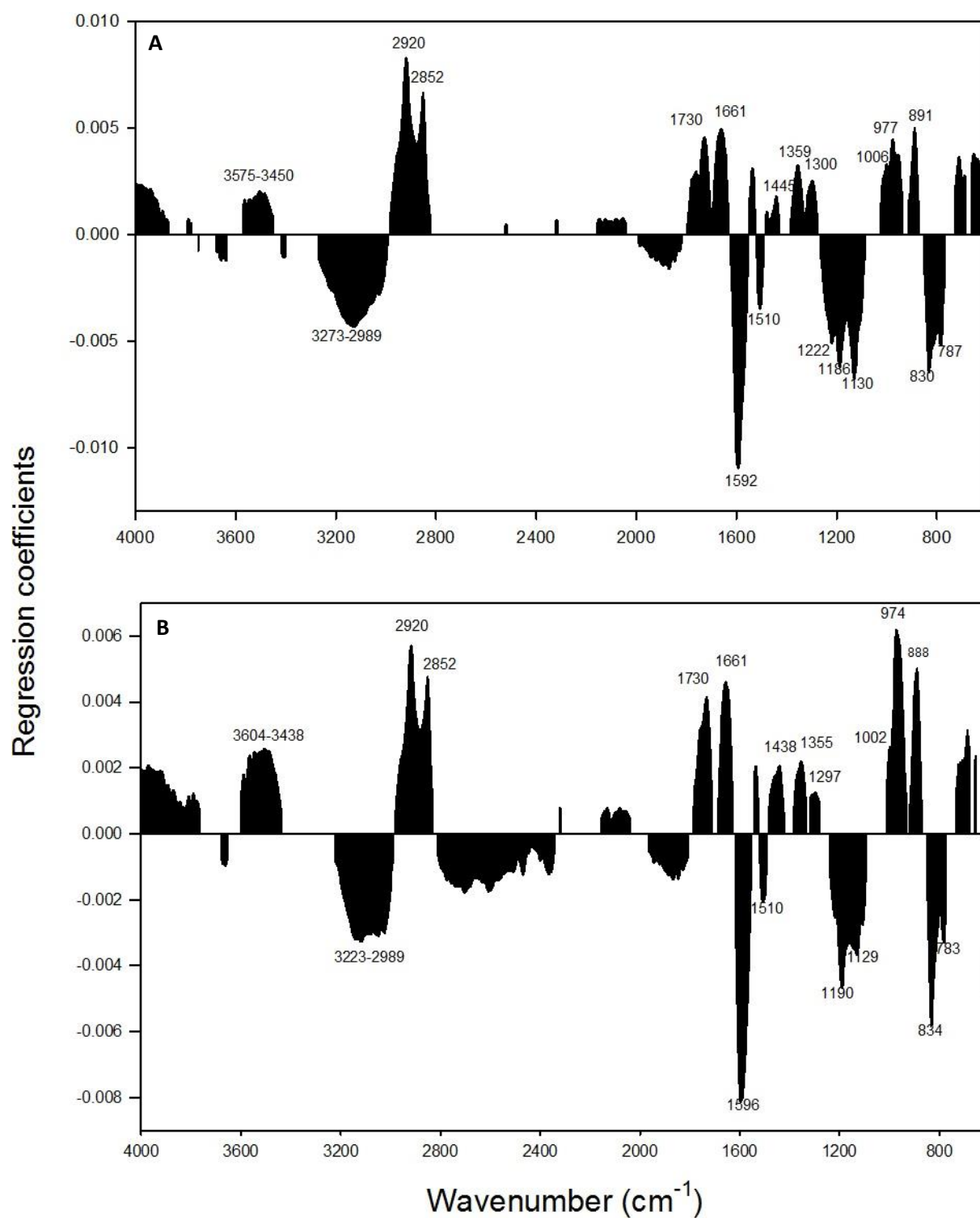

Supplement: Additional file 1: — Regression coefficients from the prediction of glucose and xylose release before the division of the calibration set into three smaller subsets. Spectral regions with a significant contribution in the prediction of glucose (A) and xylose (B) release during bioethanol production. [file 13068_2015_267_MOESM1_ESM.pdf]
